# Comparing the Interface pressure distribution of the full body chest-lumbar cushion made of memory cotton with the traditional chest cushion

**DOI:** 10.1186/s12891-021-04668-w

**Published:** 2021-09-30

**Authors:** Zhiwei Zhang, Zhiqun Jiang, Ying Wu, Yu Yan, Weiqiang Chen, Yu Zeng

**Affiliations:** 1 Department of Operating Room, The First Affiliated Hospital of Nanchang University, Nanchang, China; 2 Department of Neurosurgery, The First Affiliated Hospital of Nanchang University, Nanchang, China

**Keywords:** Full body chest-lumbar cushion made of memory cotton, Traditional chest cushion, Standard lateral position, Interface pressure, Repeated-measures

## Abstract

**Background:**

Pressure injuries are common complications occurred duration hospitalization, whether the interface pressure distribution in full body memory cotton chest-lumbar cushion was superior than traditional chest cushion remains unclear.

**Purpose:**

This study aimed to compare the effects that the full body memory cotton chest-lumbar cushion versus traditional chest cushion on interface pressure.

**Methods:**

A total of 66 healthy individuals were recruited. The volunteers were placed in the left lateral position and left armpit and iliac spine pressure and level of comfort were measured. Group differences were assessed using the paired *t*-test or Wilcoxon test according to data distribution. Additionally, multivariate regression analysis was applied to determine the potential role of sex, age, and body mass index on left armpit and iliac spine pressure and overall comfort.

**Results:**

Compared with the traditional chest cushion, we noted that the full body chest-lumbar cushion made of memory cotton was associated with less pressure on the left armpit (38.17 ± 10.39 mmHg vs. 67.93 ± 14.67 mmHg, respectively; *P* < 0.0001) and iliac spine (43.32 ± 13.70 mmHg vs. 50.77 ± 20.94 mmHg, respectively; *P* = 0.0004). Moreover, we noted that the overall comfort with the memory cotton chest-lumbar cushion was higher than that with the traditional chest cushion (8.48 ± 1.08 vs. 6.36 ± 1.45, respectively; *P* < 0.0001). Finally, the multivariate regression analyses found iliac spine pressure could affect by sex (*P* = 0.0377) and body mass index (*P* = 0.0380).

**Conclusions:**

The full body chest-lumbar cushion made of memory cotton had beneficial effects on left armpit and iliac spine pressure and on comfort. These findings should be applied to future clinical practice.

## Introduction

Pressure ulcers, defined as localized tissue breakdown of the skin and adjacent tissue, are caused by high pressure and mechanical force on the bony prominences [6, 7, 13]. Patients with pressure ulcers, which are associated with poor quality nursing care, have considerable pain and discomfort at the wound sites [3, 12]. Nowadays, the prevalence of pressure ulcers is related to economic factors and varies worldwide [4, 5, 14]. To place patients in backrest positions (< 30°) could reduce the risk of pressure ulcers because of the most common sites of pressure ulcers include the sacrum, coccyx, heels, and the hips, but other sites such as the elbows, knees, ankles, and the back of the cranium are also potential areas of concern [1]. Pressure ulcers should be managed and prevented since they are difficult to treat, increase the cost of hospitalization, and can lead to serious complications.

The redistribution of pressure plays an important role in improving the progression and prognosis of pressure ulcers [16]. Pressure relief cushions are commonly used in the clinical setting and are classified according to the materials they are made of, which affect the loading surface, contact area, and overall interface pressure [10]. The memory cotton full body chest-lumbar cushion has the potential to augment pressure relief through an increase in the total area of stress, which leads to a reduction in pressure at the stress points; however, this is not confirmed in clinical practice. Therefore, this study aimed to compare the interface pressure and comfort of the full body chest-lumbar cushion made of memory cotton with the traditional chest cushion by using a repeated-measures approach.

## Methods and materials

### Study subjects

The Ethics Committee of the First Affiliated Hospital of Nanchang University approved this study (No. 2019–103), and the STROBE Guideline was used for conducting and reporting this study. Healthy staff in our hospital and volunteers were recruited from December 2019, but those individuals who could not maintain the left lateral position for 5 min were excluded. Written informed consent for publication was obtained from all participants. A total of 66 volunteers, which included surgeons, anesthesiologists, operating room nurses, bedside nurses, nurse practitioners, and others were recruited. The classification of body mass index (BMI) was based on the Ministry of Health of China guidelines (BMI < 18.5 kg/m^2^ was considered underweight, 18.5–24.0 kg/m^2^ was normal weight, 24.0–28.0 kg/m^2^ was overweight, and > 28.0 kg/m^2^ was considered obese). The common sites of intraoperative pressure injuries were the armpits and the iliac spine, and pressure injuries occurring in the shoulders were relatively rare according to feedback from the researchers in the pressure ulcer group in the operating room. A total of 66 volunteers were thus recruited, and the pressure data at the left armpit and iliac spine were collected according to clinical practice.

### Cushion features

**Fig. 1:**
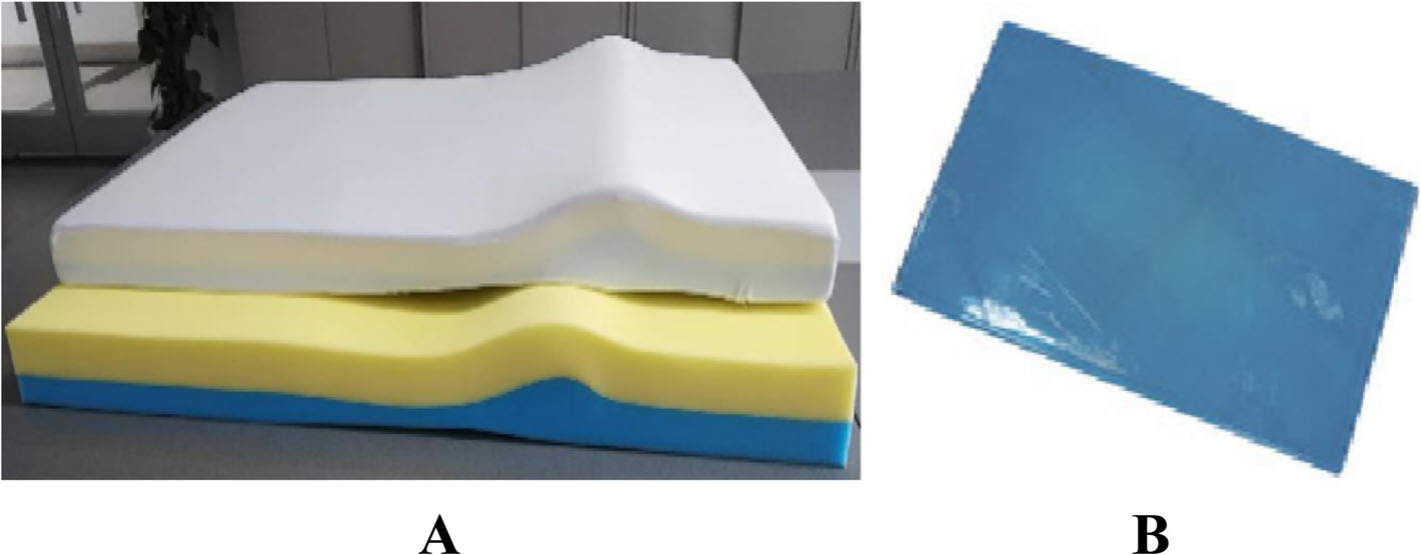
The details of the full body chest-lumbar cushion and traditional chest cushion

The full body chest-lumbar cushion was made of memory cotton, at 140 cm × 45 cm × 8 cm for length, width, and height, respectively, to ensure the memory cushion could cover the operating bed at least 10 cm from the alar and follow the physiological curve of the waist owing to the 10 cm wide arch from the lateral edge of the underarm. The traditional chest cushion was 115 cm × 45 cm × 1.5 cm, made of polymeric polyurethane gel, and had a soft texture. The details of full body chest-lumbar cushion and traditional chest cushion are shown in Fig. 1.

### Study procedure

All volunteers were uniformly placed in the standard left lateral position, and a repeated-measures approach was applied. This placement was conducted by 4 operating room nurses who were trained together. The volunteers in both groups were placed in the standard lateral position by 2 nurses each with either the traditional chest cushion or the full body chest-lumbar cushion made of memory cotton. Specifically, the placement method for the standard left lateral position is as follows: put the patient in the lateral position, then place the head pillow under the head, level with the lower shoulder height to ensure the cervical vertebra remains in a horizontal position, then place the chest cushion 10 cm below the armpit. The flexed upper limb on the operative side is then placed in an adjustable arm bracket with the distal joint slightly lower than the proximal joint. The lower limb is abducted on the hand plate, and the distal joint is placed higher than the proximal joint to maintain the natural extension of the chest. Shoulder joint abduction is maintained at no more than 90°, and the two shoulders are secured to the operating table at 90°. The ventral baffle is used to support the pubic symphysis, the dorsal baffle is used to secure the sacral or scapular area, and patients are then maintained in a 90° lateral position. The natural flexion of both lower limbs is kept at about 45°, placed separately, with both legs in the running posture flexion position. The upper lower limb is supported with a support pad between the legs, and the lower leg and both upper limbs are fixed with restraint straps. The Tractilus Pressure Mapping System (Tekscan, Boston, MA, USA) includes a full-body pressure mapping pad and computer software system. The size of the sensor pad is 90 cm × 200 cm, and it consists of 1728 pressure sensing points that measure a range up to 5 PSI. In this study, the Tractilus Pressure Mapping System, which gently lifts and lowers the volunteer after positioning, was in contact with the body of the volunteer directly to reduce the interference caused by excessive folding of the pressure sensing pad during positioning.

### Outcome measurements

For the volunteers maintaining the standard left lateral position, pressure on the left armpit and iliac spine were collected if the pressure sensing system data were stable after 5 min. The comfort level was assessed by using the visual analogue scale (VAS), which measured the subjective experience of the volunteers. For this scale, a horizontal line of 10 cm is drawn on a piece of paper, with one side of the line marked 0, indicating extreme discomfort, and the other side marked 10, suggesting comfort.

### Statistical analysis

The values of age, height, weight, and BMI were reported as the mean ± standard deviation if they were normally distributed. Otherwise, the data were presented as the median and interquartile range. Moreover, the distribution of sex was displayed as a number and proportion. The paired *t*-test and Wilcoxon test were used to compare the subjective comfort and the left armpit and iliac spine pressure in the full body, memory cotton chest-lumbar cushion group with the traditional chest cushion group according to data distribution. The multivariate regression analyses were performed to explore the potential role of sex, age, and BMI on left armpit and iliac spine pressure and comfort. IBM SPSS Statistics for Windows, version 19.0 (SPSS 19.0) was used for all analyses in this study.

## Results

### Characteristics of study subjects

The characteristics of the recruited volunteers are presented in Table 1. Of the 66 healthy volunteers, 50 were female, and the remaining 16 were male. The mean age of the volunteers was 32.18 years, the mean height was 163.62 cm, mean weight was 61.92 kg, and the mean BMI was 23.14 kg/m^2^.

**Table 1:** The included participants’ characteristics

Variables	Value (*n* = 66)
Sex [n(%)]	
Female	50 (75.76)
Male	16 (24.24)
Age (years) [Mean(SD)]	32.18 (8.09)
Height (cm) [Mean(SD)]	163.62 (5.82)
Weight (kg) [Mean(SD)]	61.92 (10.70)
BMI (kg/m^2^) [Mean(SD)]	23.14 (4.05)

### Left armpit and iliac spine pressure and subjective comfort

**Fig. 2:**
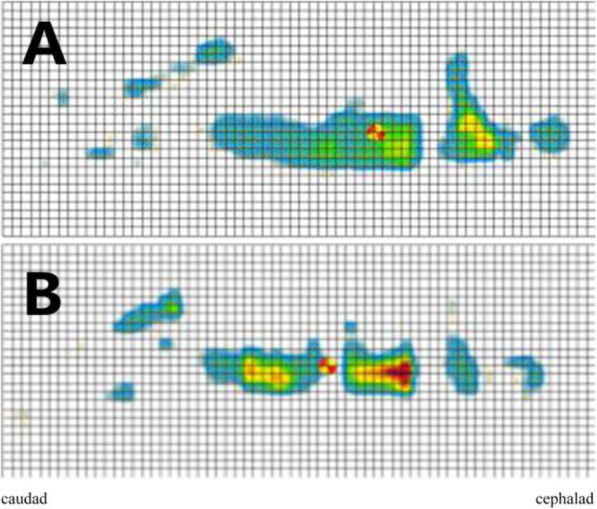
Four quadrants of interface pressure mapping in the full body chest-lumbar cushion made of memory cotton group (**A**) and the traditional chest cushion group (**B**). Red indicated high interface pressure, yellow indicated moderate interface pressure, and blue indicated low interface pressure

The interface pressure mapping of the full body chest-lumbar cushion made of memory cotton and the traditional chest cushion groups are presented in Fig. 2. Table 2 summarizes the pressure on the left armpit and iliac spine and subjective measurements of comfort for the volunteers in both groups. We noted that the full body chest-lumbar cushion made of memory cotton was associated with less left armpit pressure compared with the traditional chest cushion (38.17 mmHg [10.39] vs. 67.93 mmHg [14.67], respectively; *P* < 0.0001), less iliac spine pressure (43.32 mmHg [13.70] vs. 50.77 mmHg [20.94], respectively; *P* = 0.0004), and a higher level of comfort (8.48 [1.08] vs. 6.36 [1.45], respectively; *P* < 0.0001).

**Table 2:** Characteristics of left armpit and iliac spine pressure and overall comfort

Variable	Overall memory function chest - lumbar cushion	Traditional chest cushion	Difference between groups	*P* value
Left armpit^a^				
N(Missing)	66 (0)	66 (0)	66 (0)	< 0.0001
Mean(SD)	38.17 (10.39)	67.93 (14.67)	−29.76 (15.30)	
Median	36.22	67.33	−28.58	
Q1,Q3	31.60,42.59	57.63,77.62	−39.08,-20.85	
Min,Max	22.88,66.60	42.59,118.62	−77.29,3.78	
Spina iliace^b^				
N(Missing)	66 (0)	66 (0)	66 (0)	0.0004
Mean(SD)	43.32 (13.70)	50.77 (20.94)	−7.45 (19.58)	
Median	39.56	45.04	−7.00	
Q1,Q3	35.01,46.51	38.04,53.97	−15.29,1.52	
Min,Max	23.38,98.36	3.92,123.98	−64.85,38.80	
Comfort^b^				
N(Missing)	66 (0)	66 (0)	66 (0)	< 0.0001
Mean(SD)	8.48 (1.08)	6.36 (1.45)	2.13 (1.42)	
Median	9.00	6.50	2.00	
Q1,Q3	8.00,9.00	5.00,7.00	1.00,3.00	
Min,Max	4.00,10.00	0.00,9.00	−1.00,8.00	

^a^paired t test; ^b^Wilcoxon test

### Multivariate regression analysis

The results of multivariate regression analyses for the role of sex, age, and BMI on left armpit and iliac spine pressure and overall comfort are listed in Table 3. First, we noted that the left armpit pressure was not affected by sex (*P* = 0.5342), age (*P* = 0.2283), or BMI (*P* = 0.8351). Second, sex (*P* = 0.0377) and BMI (*P* = 0.0380) are significantly associated with iliac spine pressure, while age did not affect iliac spine pressure (*P* = 0.4235). Third, sex (*P* = 0.5870), age (*P* = 0.3698), nor BMI (*P* = 0.5708) were not associated with comfort level.

**Table 3:** Results of the multivariate regression analyses

Outcomes	Variables	β	SE	SR coefficient	t value	*P* value
Left armpit	Intercept	−37.914	13.313	0.000	−2.848	0.0060
	Sex	−2.815	4.503	−0.079	−0.625	0.5342
	Age	0.290	0.238	0.153	1.217	0.2283
	BMI	0.101	0.482	0.027	0.209	0.8351
Iliac spine pressure	Intercept	−29.280	16.201	0.000	−1.807	0.0756
	Sex	−11.634	5.479	−0.257	−2.123	0.0377
	Age	0.234	0.290	0.096	0.806	0.4235
	BMI	1.243	0.586	0.257	2.120	0.0380
Comfort	Intercept	2.361	1.243	0.000	1.899	0.0622
	Sex	−0.230	0.420	−0.070	−0.546	0.5870
	Age	0.020	0.022	0.114	0.903	0.3698
	BMI	−0.026	0.045	−0.073	− 0.570	0.5708

## Discussion

The progression of pressure ulcers is significantly correlated with prolonged mechanical loading, which can lead to inadequate blood supply and reperfusion injury through the breakdown of skin and underlying tissues [15]. In this study, in which 66 healthy volunteers were recruited from one hospital, the full body chest-lumbar cushion made of memory cotton was associated with less pressure on the left armpit and iliac spine. Moreover, the comfort level in this group was significantly higher than that in the traditional chest cushion group. These results suggest that the full body chest-lumbar cushion made of memory cotton should be used in clinical practice, especially for those placed in the standard lateral position.

Tissue ischemia and hypoxia are significantly related to the perfusion of capillary blood to the tissues if the continuous vertical pressure exceeds the normal capillary pressure (16–32 mmHg) [11]. A study conducted by Gao et al. found that irreversible damage to tissues could occur if local tissues are exposed to a pressure of 70 mmHg for > 2 h [2]. Moreover, Pieper et al. found that if local blood flow is obstructed, a subcutaneous tissue ischemia block can occur when the external pressure exceeds 32 mmHg [9]. These authors suggest, therefore, that long-term low pressure can cause more severe tissue damage than short-term high pressure. The patients in this study experienced maximum pressures on the axilla and iliac spine that were greater than 32 mmHg, which suggests that patients undergoing surgery can suffer irreversible damage if compression at this level is prolonged. Since the gravity acting on the volunteers was constant, and the contact surface area in the standard lateral position is smaller than that in other supine positions, the stress points in this position are exposed to high, intense pressure. Therefore, improving the nursing staff’s knowledge about pressure ulcers, developing prevention and position placement plans, and using local protection strategies could prevent the occurrence and progression of pressure ulcers.

We noted pressure on the alar and iliac spine was higher than that on other sites. One potential reason for this could be the shoulder and iliac spine in most volunteers contacted the surgical bed first when the body was in the standard lateral position. The concave part of the waist is in a suspended state, which reduces the pressure on these parts due to the physiological curve that exists after an axillary cushion is placed according to the requirements for standard lateral position. This causes the body pressure to be focused on the armpits and iliac spine, which is associated with a high risk of pressure injury. A study conducted by McInnes et al. found that pressure could be reduced by changing the distribution of the local pressure in the body, which could prevent the progression of intraoperative pressure ulcers [8]. Moreover, we noted full body chest-lumbar cushion made of memory cotton had beneficial effects on left armpit and iliac spine pressure and on comfort as compared with traditional chest cushion. The potential reason for this could be the full body chest-lumbar cushion made of memory cotton was used since it could conform to the physiological curve in standard lateral position. Our study designed as self-controlled and the individuals’ weight was unchanged. Furthermore, the design of the full body chest-lumbar cushion with the girth joint could support the concave part of the waist as well. The use of this cushion could increase the contact area of patients, causing the force of gravity to be more equally distributed and reducing the pressure on the local stress points. Furthermroe, the type of material and texture of cushion between full body chest-lumbar cushion made of memory cotton and traditional chest cushion could affect the peak pressure because of softness between groups was different [8].

Several limitations of this study should be acknowledged: (1) most of the recruited volunteers were female owing to the fact that data was collected from the operating room; (2) the BMI in most of the recruited volunteers was normal, and therefore may not reflect the intrinsic results in clinical practice; (3) this study analyzed only two contact areas in the standard lateral position and pressure at other sites was not collected; and (4) stratified results according to volunteers’ characteristics were not conducted owing to the small number of recruited volunteers.

The findings of this study demonstrated that the full body chest-lumbar cushion made of memory cotton was associated with lower pressure on the left armpit and iliac spine compared with the traditional chest cushion. Moreover, the comfort of those in the full body chest-lumbar cushion made of memory cotton group was higher than that in the traditional chest cushion group. Further studies that recruit volunteers who have a broad range of characteristics and compare pressures at various sites stratified by the characteristics of the volunteers should be undertaken.

## Data Availability

The datasets generated during and/or analyzed during the current study are available from the corresponding author on reasonable request.

## Abbreviations

BMI: Body Mass Index
VAS: Visual Analogue Scale
